# Microglial lipid metabolism and epigenetic regulation: Emerging targets for spinal cord injury therapy

**DOI:** 10.1016/j.neurot.2026.e00891

**Published:** 2026-03-30

**Authors:** Nicolas Guérout

**Affiliations:** Université Paris Cité, CNRS UMR8003, Saints-Pères Paris Institute for the Neurosciences, F-75006, Paris, France

**Keywords:** Spinal cord injury, Lipid metabolism, Microglia, Epigenetic regulation, Glial scar, Innovative therapy

Spinal cord injuries (SCI) most often result from traumatic events such as road traffic accidents, falls, or acts of violence [1]. These injuries have severe consequences and frequently leave affected individuals with permanent disabilities, leading to loss of motor, sensory, and autonomic functions. Depending on the level and severity of the lesion, patients may experience paraplegia or tetraplegia, which can be either complete or incomplete [2].

From a cellular and molecular perspective, SCI involves two successive phases. The initial mechanical trauma defines the primary injury, while the cascade of events triggered by this trauma constitutes the secondary injury. This secondary phase begins within seconds after the initial insult and can extend over several months or even years. It is characterized by a series of events including destruction of the spinal parenchyma, hemorrhage, death of neurons and glial cells at the lesion site, increased permeability of the blood–spinal cord barrier, infiltration of peripheral immune cells, and activation of endogenous cells, notably astrocytes and microglia [3]. Together, these processes lead to the formation of a scar.

This scar, initially described as a “glial scar,” is in fact composed of multiple cell populations including astrocytes, fibroblasts, microglial cells, and macrophages [4]. It was originally considered to be inhibitory to axonal regeneration. However, its role is now recognized as more complex, as it can also secrete factors permissive for axonal regrowth and contributes to confining inflammation within the lesion core, thereby preventing its spread to the surrounding spared tissue [5].

It is within this scientific context, focusing on the spinal scar, that the work of Qiao et al. described in this issue of Neurotherapeutics takes place [6].

In this study, the authors first focused on microglial cells and their lipid metabolism, particularly cholesterol metabolism. They further demonstrated that pharmacological modulation of microglial metabolic pathways reduces spinal scar formation and promotes functional recovery in treated animals. To address these questions, the authors used a mouse model of spinal cord compression injury and applied state-of-the-art techniques, including single-cell RNA sequencing (scRNA-seq) and single-cell ATAC sequencing (scATAC-seq), which enables the genome-wide assessment of chromatin accessibility across individual cell types. Using scRNA-seq analyses, they first showed that microglia represented the cell population most strongly expanded following injury. Functional enrichment analyses further revealed a significant dysregulation of cholesterol metabolism in these cells, both at 7 days and 2 months after SCI. This metabolic alteration was accompanied by an accumulation of cholesterol within microglial cells. Subsequently, by combining scATAC-seq and immunohistochemical approaches, the authors identified changes in chromatin accessibility associated with regulatory regions marked by the histone modification H3K27ac, particularly in astrocytes and, more prominently, in microglial cells. Finally, ChIP-qPCR analyses demonstrated that H3K27ac directly contributes to the transcriptional regulation of genes involved in cholesterol metabolism.

Following these experiments, the authors sought to investigate the role of H3K27ac *in vivo* after SCI. To this end, they used the acetyltransferase inhibitor L002 to reduce H3K27ac levels. They showed that administration of L002 decreased H3K27ac expression in microglial cells, which in turn modulated their lipid metabolism, notably by reducing cholesterol accumulation within these cells. Furthermore, this reduction in cholesterol accumulation was associated with decreased expression of pro-inflammatory cytokines. At the tissue and functional levels, the authors demonstrated that L002 treatment modulated spinal scar formation, notably by reducing the fibrotic core and astrocytic reactivity, as well as by decreasing microglial activation. Importantly, this modulation of the scar was accompanied by increased neuronal and oligodendrocyte survival and reduced post-traumatic demyelination. These histological changes were correlated with improved functional recovery, as demonstrated by several motor behavioral tests.

Altogether, this study highlights the central role of microglial cells, and particularly their lipid metabolism, in spinal scar remodeling and functional recovery following SCI. This work clearly illustrates the link between understanding the cellular and molecular mechanisms that occur after SCI and the identification of new therapeutic strategies. It also emphasizes the key role that OMICS technologies now play—and will continue to play—in such discoveries, as exemplified here by the use of scRNA-seq, scATAC-seq, and ChIP-qPCR approaches. Indeed, as described above, the authors first took advantage of these innovative technologies to characterize previously unrecognized cellular and molecular mechanisms. These findings are then used to identify pharmacological agents capable of modulating the pathways involved, before subsequently testing the therapeutic potential of these agents *in vivo* in a preclinical model.

This study also gains particular significance in light of another recent study highlighting the fundamental role of microglial cells in SCI. In particular, McCallum et al. recently published a study emphasizing the role of microglia and their lipid metabolism in the clearance of myelin debris following SCI [7]. In this work, the authors showed that within the peri-lesional area there exists a population of astrocytes—referred to as *lesion-remote astrocytes*—that regulates microglial activity adjacent to the lesion. These astrocytes, characterized by the expression of CCN1, interact with microglial cells and promote the clearance of myelin debris generated by the degeneration of descending motor tracts after injury [7]. Interestingly, this study also relies on advanced OMICS approaches. These methods first revealed a differential spatial distribution of astrocyte populations within the spinal cord under physiological conditions as well as after SCI. They then enabled the identification of the key role of these lesion-remote astrocytes in myelin debris clearance and lesion remodeling after SCI through their interaction with microglial cells.

Although these two studies pursue distinct objectives, they both converge in highlighting the central role of microglial cells and their modulation in post-injury tissue remodeling following SCI. This point is particularly important. Although the central role of these cells is widely recognized, whether microglia exert beneficial or deleterious effects after SCI remains highly debated [8,9]. Together, they further underscore the potential importance of targeting microglial biology for future therapeutic strategies. Modulating these cells could represent a promising approach not only for the treatment of SCI, but also for other demyelinating disorders of the central nervous system, such as Multiple Sclerosis.

## Author contributions

N.G. wrote the article.

## Declaration of competing interest

The authors declare that they have no competing interests.
